# Maternal Schistosomiasis: Immunomodulatory Effects With Lasting Impact on Allergy and Vaccine Responses

**DOI:** 10.3389/fimmu.2018.02960

**Published:** 2018-12-18

**Authors:** Matthew Lacorcia, Clarissa U. Prazeres da Costa

**Affiliations:** Department of Medicine, Institute for Medical Microbiology, Immunology, and Hygiene, Technical University of Munich, Munich, Germany

**Keywords:** schistosomaisis, immune regulation, developmental immune modulation, maternal infection, allergy, vaccines

## Abstract

Early exposure to immune stimuli, including maternal infection during the perinatal period, is increasingly recognized to affect immune predisposition during later life. This includes exposure to not only viral and bacterial infection but also parasitic helminths which remain widespread. Noted effects of helminth infection, including altered incidence of atopic inflammation and vaccine responsiveness, support further research into the impact these infections have for skewing immune responses. At the same time, despite a sea of recommendations, clear phenotypic and mechanistic understandings of how environmental perturbations in pregnancy and nursing modify immune predisposition and allergy in offspring remain unrefined. Schistosomes, as strong inducers of type 2 immunity embedded in a rich network of regulatory processes, possess strong abilities to shift inflammatory and allergic diseases in infected hosts, for example by generating feedback loops that impair T cell responses to heterologous antigens. Based on the current literature on schistosomiasis, we explore in this review how maternal schistosome infection could drive changes in immune system development of offspring and how this may lead to identifying factors involved in altering responses to vaccination as well as manifestations of immune disorders including allergy.

## Introduction

Growing research continues to expose the delicate immunological balance at the fetomaternal interface, its vulnerability to perturbation by maternal infections, and the subsequent effects of such disruptions on immune development and responses later in life. Bacterial infections such as listeriosis remain key concerns that threaten healthy pregnancy (1). Perinatal viral and bacterial infections have been shown to impact normal development and potentially lead to behavioral shifts in later life (2). Attention has also been given to perinatal helminth infections, which is understandable considering that schistosome infections alone affect over 200 million people, including pregnant women. This means that a conceivably large number of children are born having either direct or indirect gestational exposure to helminthic parasites and their products, or maternal mediators produced in reaction to infection such as cytokines. Throughout human evolutionary history such infections were likely to be even more prevalent, and as such, are intimately linked to the pressures under which the modern immune system evolved. More generally, the interplay between infections and the inflammatory responses they elicit present significant events that feed into immunological memory, and complement genetic components providing modulatory effects through the lifetime of immunological challenges that shape our immune predispositions. This includes modulatory immune processes not only induced by microbes and parasites to ensure their survival, but also as feedback responses to dampen inflammatory sequelae and additional damage resulting from infection. It therefore remains important to evaluate how these exposures may be required in instructing tolerogenic states that appear to be lacking or imbalanced in not only the disproportionate responses that comprise allergy (3), but also along the axis of immune surveillance between triggering autoimmune disease and allowing the evasive survival of cancers (4).

Much of the epidemiological impact of helminth infections with strong immunomodulatory effects has been extensively reviewed in Feary et al. (5), Janssen et al. (6), Wammes et al. (7), including the range of effects that *Schistosoma mansoni* infection, or exposure to its released products, live or dead eggs, soluble egg antigen (SEA) extracts, or even recombinantly expressed schistosome-derived proteins have upon inflammatory and autoimmune diseases. This current review instead aims to place these and more recent findings within the context of perturbations during immune ontogenesis, where much research has thus far focused on bacterial and viral infections. By making an overview of mechanistic lessons learnt from such approaches using other infection modalities, we identify avenues for future research addressing potential immunoregulatory processes induced via exposure to helminth infection, which may help to develop understandings of how these may be incorporated for beneficial interventions to fine tune cellular responses to vaccines and control immune disease.

## Regulation Through “Immune Education”: Microbial Exposures and Other Immunological Challenges During Key Phases of Early Life

The latter half of the 20th century was marked by a dramatic rise in the incidence of immune disorders and diseases of chronic inflammation. Whether autoimmune conditions, such as type 1 diabetes or multiple sclerosis, or allergic conditions such as asthma, atopy, and atopic dermatitis (eczema), or chronic pathologies like type 2 diabetes, metabolic syndromes or inflammatory bowel disease, these conditions are tied through shared underlying inflammatory processes (8) and the loss of immune regulation, such as mediated through regulatory T cells (9). This stark upsurge over such a relatively short time period, evolutionarily, implicates environmental factors in the etiology, which include dietary changes, psychosocial stress, xenobiotics and pollutants, alterations to the commensal microbiome, and changes to infectious burden (10, 11). Concerning the latter, sanitary practices have led to a strong decrease in gastrointestinal and fecal-oral parasitic infections, and the association of this with hyperactive immune responses led to the formation of the so-called “hygiene hypothesis” (12). The “old friends” expansion of this theory supposes that human evolution under the burden of such infectious agents, many of which dampen the immune system in order to ensure their survival within their host, produced a hypervigilant immune system, which, lacking these dampening checkpoints in its more urbanized, sanitized form, is free to cause immune-mediated pathology (13). The question then arises whether the mechanisms used by these organisms, parasites, for example, to dampen the immune system, can inform us not only about the etiology of immune-mediated diseases, but also perhaps provide potential avenues through which to modulate the immune system, prevent overly reactive states, and cool-down inflammatory predispositions.

Much work over the past decades has explored this interplay between infection and immune disorders. Specific immunoregulatory agents such as bacteria and other microbes have been found in farming environments, supporting inverse associations between early life farm exposure and allergy (14). Transgenerational effects from maternal exposures include the case of *Acinetobacter lwoffii* F78, a high LPS content, atopy-protective bacterium from farming environments (15), which was shown to upregulate suppressor molecules in lung epithelial cells through a negative feedback loop and thus to dampen airway inflammation, an effect also recreated by LPS exposure alone (16). Perinatal application of this bacterium in mouse models has been shown to render offspring hyporesponsive to experimental airway inflammation, mediated at least in part through providing increased maternal Toll-like receptor (TLR) stimuli (17). Follow-up work identified epigenetic alterations to the T cell compartment in such perinatally-exposed offspring, where robust permissive signatures on IFN-γ promoters in helper T cell populations inhibit experimentally-induced allergic airway inflammation (17, 18). In another recently published model, maternal infection with *Helicobacter pylori* also decreased offspring responsiveness to experimental airway inflammation induction, here mediated through induction of a regulatory T cell phenotype in the offspring and recreated through transmaternal application of the key *H. pylori* immunomodulatory compound VacA (19). Increased interferon (IFN)-γ levels, one outcome of typical viral or bacterial exposures, have been shown in a mouse model through direct, controlled administration during pregnancy to interrupt the progression of allergic phenotypes (20). However, much work on the effects of viral infection during pregnancy have highlighted the deleterious effects potentially played by such maternal immune activation. Mouse models of viral infection during gestation using poly I:C as an analog for viral double stranded (ds)RNA have demonstrated mostly adverse effects on offspring behavior triggered by the increase in pro-inflammatory cytokines, such as IL-6 and IL-17A (21). More recently, these effects in offspring were also replicated using preconception microbiota transfer from such poly I:C exposed mice, highlighting a role for microflora in regulating and mediating these effects (22).

Exposure to a range of environmental factors, such as maternal and paternal stress, diet, and other practices (such as antibiotic usage) that effect microbiota during early life, have demonstrated impact on the development of conditions including immune disorders, allergy, as well as neurodevelopmental disorders such as autism and schizophrenia (23, 24). Maternal factors beyond infection, including toxins, stress, and obesity, can impact inflammation and have been implicated in altered immune and behavioral outcomes in offspring (25). In general terms of immune development, the impressionable time window of the prenatal period and the first “1,000 days” presents numerous challenges to the developing immune system that can either push it toward the development of non-communicable diseases (such as inflammatory conditions) or toward increased states of maturation associated with healthy responses (26). In fact, such alterations to maturation of the immune system are associated with switches in immune response that highlight the age and life-stage specificity of appropriate immune responses. For example, although increased IL-13 is a hallmark of allergic and atopic conditions, faulty or reduced fetal and neonatal mitogen-induced IL-13 responses upon stimulation of cord blood mononuclear cells were associated with a family history of atopy, with increased responses therefore taken as a potential indicator of healthy immune maturation (27). However, increased IL-13 production from stimulated CD4^+^ T cells isolated from cord blood has also been associated with increased rates and severity of atopy and eczema (28). Additionally, following early life farm exposure, lower incidence of allergy was associated with increased levels of FoxP3^+^ regulatory T cells (Tregs) in peripheral blood at 4.5 years of age, and yet with decreased levels of such Tregs by 6 years of age (29), indicating age-related fluctuations in these features. These signify the dynamic processes involved in healthy immunoregulation, and the complexity of regulatory processes that probably underlie immune phenomena resulting from altered maternal exposures. The following sections review studies on how perinatal helminth infection can serve to induce similar end-point shifts in allergic responses in offspring as seen with protective microbial exposures, most likely also through complex networks of immunological feedback. What sets helminth infections apart from bacterial and viral infections, however, is that they induce predominantly type 2 immune responses and employ complex strategies to avoid clearance and attain chronicity. So, while such studies bear similarities to modalities of microbial exposure-induced regulation, and potentially similar effects to the disrupting immunological stimulation evoked by viral infection, there are also important points of difference.

## Immunomodulation Through *Schistosoma mansoni* Infection

The “old friends” expansion of the hygiene hypothesis includes parasitic helminth infections within the range of exposures which can alter inflammatory disease and allergy. The strong type 2 responses which characterize chronic *Schistosoma mansoni* infections are largely stimulated by the parasite eggs and their soluble molecules, that also induce strong autoregulation to dampen inflammatory responses (30–33). Such chronic infections have a demonstrated effect on suppressing bystander immune responses which has been extensively reviewed (34–36). Some of the strongest data for associations between helminth infection in humans and immunomodulation pertain to reduced allergic skin-prick testing responses. For example, a Brazilian study of people with heavy *S. mansoni* infections were on average 5-fold less reactive to skin-tests with allergens compared to matched uninfected individuals from the same region (37), further assessed in Feary et al. (5). More recently, a study on Ethiopian rural migrants moving to urbanized areas in Israel found they had less allergy if they harbored an *S. mansoni* infection, and displayed significant increases in allergy if they underwent antihelminthic treatment (38). On a mechanistic level, responses to Derp1 (a major dust allergen) in *S. mansoni*-infected asthmatics, compared to uninfected asthmatics, showed reduced allergen-induced IL-4 and IL-5 levels from PBMCs, while the allergen-induced IL-10 production was higher from these infected individuals (39, 40). Murine studies also support observations that *S. mansoni* reduces severity of pathology resulting from co-infections such as malaria, including progression to cerebral malaria (41), as well as inflammation from autoimmune processes (42).

Concerning the underlying mechanisms of such interactions in immunological terms, the classical paradigm of imbalanced type 1/type 2 immune responses might not be applicable to the effects that schistosome infection have on allergic type 2 responses, possibly due to their very dynamic immune phases and presentations. Indeed, the strong suppressive effects of helminth infection upon allergy appear to be in chronic phases where type 1 responses have largely subsided, and instead are replaced by an immune state characterized by modified type 2 immune processes coupled to suppressive, regulatory aspects such as regulatory and IL-10-producing T and B cells (31, 43). As such, rather than simply modifying the type 1/type 2 balance, the persistent challenge with immune stimuli, such as a broad spectrum of diverse microbiota, infections, and parasites, induces a regulatory network that is more equipped or primed to effectively manage challenge with novel stimuli, such as potential allergens. The apparent paradox of helminths, as strong type 2 stimuli, reducing atopic inflammation, despite association with increased sensitization to allergens such as allergen-specific IgE levels (44), but with the presence of increased IL-10 levels, invites investigation into regulatory feedback loops induced by schistosomes that overshoot to suppress wider inflammatory responses to other antigens (45). Studies on infected mice have found reduced cytokine production and lowered T cell proliferation to heterologous antigens to be largely dependent on parasite infection-related increased IL-10 levels (46). Further, these effects are associated with the induction of regulatory phenotypic changes in immune cells, including alternative activation of macrophages, induction of myeloid derived suppressor cells, tolerogenic phenotypes in dendritic cells, and regulatory IL-10 producing T and B cells, as reviewed in Wammes et al. (7).

## Effects of Maternal Schistosomiasis on Vaccine Efficacy in Offspring

Additionally, the suppressive bystander effects from the type 2 and regulatory immune responses present during chronic helminth infections are associated with altered vaccine efficacy, including effects of chronic schistosomiasis on reducing protective type 1 responses raised against tetanus vaccines (47) and Hepatitis B (48), reflecting the dampening effects of *Trichuris trichiura* on antibody titers obtained from anti-malarial vaccines (49). As such, discovery of effective vaccines therefore continues to be a particular concern for communities where helminths are endemic (50). Murine models have also displayed reduced vaccine efficacy following schistosoma infection, for example, against *Mycobacterium tuberculosis* (51), reducing type 1 responses typified by IFN-γ and instead increasing type 2 responses. Recently, experimental murine *S. mansoni* infection inhibited effective cellular and antibody responses against novel HIV vaccines, with even the presence of schistosoma eggs alone (as a result from de-worming) able to reduce humoral vaccine responses (52). Nevertheless, effects of antihelminthic treatment on vaccine efficacy in human cohorts remains unclear (53, 54). Further, there are indications that modified responses to vaccines may be transferred to the newborn children of infected mothers (55, 56). Cohort studies, for example, have shown that developmental exposure to maternal helminth infection, with measurable effects on offspring immune priming including altered IL-10 levels in cord blood, can reduce levels of protective IgG in response to vaccination against *Haemophilus influenzae type B* and Diphtheria (57, 58). A further sign of transgenerational immunomodulation is the observation that infantile eczema is low in children of *S. mansoni*-infected mothers, and can increase upon anti-helminth treatment of mothers (59). There is also evidence that maternal schistosomes can sensitize human offspring *in utero* through altered total and schistosome-specific IgE levels, and increased signs of maturation in B cells (60, 61). As such, maternal exposures driving altered responses to homologous (i.e., schistosome) antigens could represent an effect of antigen-specific maternal tolerization, already demonstrated in mice using model antigen ovalbumin (OVA) (62). Schistosome antigens and antibodies can transfer to and persist in offspring (63, 64), and such pre-sensitization via prior exposure to acutely infected mothers was shown to alter susceptibility to subsequent infection of murine offspring (65–67). Here, acute maternofetal exposure to *S. mansoni* attenuated the pathogenesis of schistosomiasis in adult age offspring (66) indicating potential adaptive immune mechanisms of antigenic transfer to the fetus and neonates and subsequent sensitization.

## Mechanisms of Immunomodulation Through Maternal Infection

Maternal parasitic infection history has in particular been shown to have a range of potential stimulatory effects on the maturation of the developing immune system, one outcome of which is often induced tolerance, particularly regarding the homologous pathogen itself (68). For example, early-life exposure can yield tolerance to parasites such as malaria, which may also lead to increased susceptibility to the same infection in later life through suppression of antigen-presenting cell function and T cell responses (69). Interestingly, cord blood cells from neonates from geographical locations highly endemic for helminth and malarial infections show many signs of immunological maturity, including lower proportions of B cells expressing the immaturity marker CD5, and lower expression of CD27 and CD28 on CD4^+^ T cells, indicating downregulation through antigen-experience (70). Mouse models have shown that alongside the tolerogenic effects of reduced allergic inflammation severity for infected hosts during patent chronic infection, characterized by IL-10 production (33), this chronic infection in dams imprints a regulatory phenotype of suppressed allergic responses upon exposed but uninfected adult offspring (71). Multiple sources show indications that maternal schistosomiasis in murine systems reduces adaptive immune responses to antigens, but these are highly dependent upon the specific modes and time points of exposure to maternal infection during pregnancy. There are however conflicting reports of an altogether opposite effect (increased humoral response) contributed by breastmilk (72, 73) of schistosome infected murine mothers, although the effects of this may be dependent on the specific models used and timepoints employed. In fact, mating infected female mice during early patent infection (as opposed to the late chronic phase), where there is a strong increase in type 2 cytokines, yields increased allergic responsiveness in offspring (71). This strongly implicates a mechanistic role for the divergent effects of maternal cytokines and immune cell profiles during these distinct immune phases, which remains to be explored.

Although complete descriptions of the underlying changes involved in these processes are lacking, there are indications that lasting modifications modify the behavior of key immune cells involved in mounting adaptive immune responses. This includes a report of altered antigen presentation, with altered expression of co-stimulatory molecules CD40, CD80, and CD86 during vaccination-based sensitization in adulthood, although these are further complicated by divergent effects (increased or decreased co-stimulatory signaling) from *in utero* exposure vs. through nursing (74). On the other hand, initial reports indicate that the T cell compartment itself may be modified, including altered production of type 2 cytokines IL-4 and IL-5, coupled with more repressive histone acetylation patterns upon the promoters of those effector cytokines on CD4^+^ T cells from murine offspring exposed to this schistosome-induced regulatory environment *in utero* and during nursing (75), as already demonstrated to reduce responsiveness to airway inflammation (71). Consequently, bystander modulatory effects of schistosome exposures on responses to heterologous antigens (such as the ability to modify immune disease, vaccination responses, or the effects of model allergen ovalbumin in animal models) remain a key area of active investigation. The effect of maternal schistosomiasis in humans upon the effective development of antibody titres remains unclear, for example, following Hepatitis B vaccination (76), as is the role of any antibody-mediated effect in experimentally-induced allergy models (71). Similarly, its impact upon vaccine efficacy in children remains inconsistent, with recent studies contrasting previous results by showing no effect regarding diphtheria and tetanus, but reduced measles vaccine responses (77). The regulatory networks induced by *S. mansoni* infection can also impact response to viral infections, with infected mice having impaired clearance of vaccinia virus (78), and this increased persistence appears mediated by suppression of CTL activity by a macrophage-like cell induced by schistosomiasis that operates through soluble factors (79), a potential pathway that could operate in transgenerational modulation of offspring but remains unexamined. Table 1 summarizes the major findings from models exploring immunomodulation through transmaternal parasite exposure, with a focus on helminth infection.

**Table 1:** Overview of immunomodulation through maternal parasite infection.

**Parasite and model**	**Study design**	**Outcome of transmaternal exposure**	**References**
Human maternal *Wuchereria bancrofti* or *Schistosoma haematobium*	*In vitro* infant peripheral blood mononuclear cell (PBMC) assessment 10–14 months after BCG vaccination at birth	Reduced type 1 cytokine responses and increased type 2 responses to vaccine antigen	(56)
Human maternal malaria and/or multiple helminth infections	36-months follow-up after infant vaccination	Reduced IgG responses to *Haemophilus influenzae type B* and Diphtheria vaccinations	(57)
Human maternal malaria and/or multiple helminth infections	30-months follow-up after infant vaccination	Increased *in vitro* cord blood mononuclear cell (CBMC) IL-10 responses to parasite-antigen (schistosome and filaria) and later reduced vaccine-induced IgG	(58)
Human maternal *Schistosoma mansoni*	Anthelminthic treatment during pregnancy, follow-up medical assessment	Untreated antenatal infection is associated with decreased risk of infantile eczema, but anthelminthic treatment increased risk	(59)
Comparison of helminth-endemic region of Kenya compared to non-endemic USA subjects	CBMC cells assayed *in vitro* for antibody production	Spontaneous IgE production, and increased helminth-antigen induced IgE and IgG	(60)
Human maternal *Wuchereria bancrofti* or *Schistosoma haematobium*	Assessment of cord blood plasma and CBMCs	Increased sign of mature in B cells as well schistosome-specific IgE	(61)
Mouse model of acute maternofetal exposure to *Schistosoma mansoni*	Re-infection of offspring from infected mothers	Presence of schistosome-specific IgG in exposed neonates, and subsequent reduced susceptibility to infection	(66)
Human maternal *Schistosoma mansoni*	Assessment of cord blood and later of infant urine	Schistosome-specific IgG detected in cord blood, and schistosome antigens detected in infant urine up to 24 months	(64)
Human maternal *Plasmodium falciparum*	*In vitro* assessment of CBMCs	Parasite Ag-specific type 1 T cell responses reduced in cases of active infection, and instead regulatory responses, alongside reduced activation markers on APCs.	(69)
Comparison between endemic region for helminth and malaria and non-endemic region	Surface marker assessment of CBMCs	CBMCs from parasite-endemic regions show increased signs of maturity in lymphocyte populations	(70)
Murine maternal *Schistosoma mansoni* infection model	Offspring challenged with experimental allergic airway inflammation and epigenetic analysis of T cell compartment in steady state	Modulation of offspring allergic response according to maternal infection phase. Tolerogenic late chronic phase associated with epigenetic shift in CD4^+^ T cells, and subsequent cytokine production capacity	(71, 75)
Murine maternal *Schistosoma mansoni* infection model, using cross-fostering to distinguish *in utero*- from nursing-derived effects	Offspring from infected mothers challenged model ovalbumin vaccination or cognate (schistosome) infection	Differential effects from schistosome exposure through nursing (which increased humoral response) compared to *in utero* exposure (which lead to IL-10-mediated tolerizing effects, and reduced co-stimulatory signalling from APCs)	(72–74)
Human maternal *Schistosoma mansoni*	6-months assessment following infant vaccinations	No apparent effect on antibody titres	(76)
Human maternal *Plasmodium falciparum* and/or *Schistosoma mansoni*	2-years assessment following infant vaccinations	Maternal schistosomiasis was associated with reduced antibody titres in response to measles vaccine	(77)

Mechanistically, cross-reactivity between antibodies raised against *S. mansoni* egg antigens and allergic plants [notably recently discovered for peanut (80) and grasses (81)] lend weight to the possibility that the modified type 2 response may induce the suppressed immunological responses to non-related antigens with features similar to those seen in individuals treated successfully with immunotherapy. This in turn may operate by virtue of glycans shared between plants and helminths yet absent from mammals, termed as cross-reactive carbohydrate determinants (CCDs) (81). In transgenerational models, there is the further possibility of epitopes from such antigenic compounds being presented within the more tolerogenic gestational context *in utero*, or to neonates via breastmilk, highlighting how an increased range of antigen exposures during this key “window of opportunity” could have direct effects on shifting immune cell populations in offspring away from hyperresponsiveness later in life. But again, mode, duration, and context of exposure may be key in determining the balance between sensitization and tolerization. For example, studies where murine neonates (similarly within an early-life window, but post-birth) were exposed schistosome antigens found an increased predisposition toward increased inflammatory sequelae, at least in regard to later secondary contact with schistosomes (82). And yet, in other contexts, modified protocols of schistosome egg exposures beginning at a similarly early age were found to completely abrogate genetically-predisposed autoimmune disease in non-obese diabetic (NOD) mouse models (83).

In terms of altering T cell responses to heterologous antigens, schistosome oligosaccharide Lacto-N-fucopentaose III (LNFPIII) was able to effect co-cultures of dendritic cells and T cells by suppressing type 1 responses (measured by IFN-γ production) from CD4^+^ T cells without a drop in CD8^+^ activity (84). LNFPIII was shown to program dendritic cell behavior leading to altered CD40/CD40L-dependant effects on natural killer cell activation, and also to drive monocytes toward an alternatively activated phenotype (85). *In utero* exposure to such compounds may warrant further investigation as potential mechanisms for training antigen presenting cell phenotypes to drive altered T cell responses, as would echo recently described mechanisms for transmaternal exposure to immunomodulatory compounds from *H. pylori* (19). Additionally, the gestational cytokine environment appears to differ during allergy-protective acute (pre-patent) and chronic maternal phases of schistosomiasis (IFN-γ and IL-10-dominated responses, respectively) (71). Such signals may operate through divergent mechanisms to alter immune development, and whether their potential effects occur directly or indirectly, remain to be disentangled, as does their relationship to other potential specific mechanisms such as derived from immunomodulatory schistosome compounds or antigenic interactions, Even increased IL-10 itself can have differential activities upon CD4^+^ vs. CD8^+^ function (86), which may be revealed as a significant factor in, for example, fine tuning vaccine efficacy where protective immunity can be associated specifically with adequate amounts of memory T cell populations.

A final mechanistic angle for transmaternal effects of schistosomiasis that cannot be excluded at this point is modification of the microbiome and metabolome. Schistosomes do not inhabit the intestinal lumen, as do other helminths such as the nematode *Heligmosomoides polygyrus*. However, their relationship to gut inflammation, including as driven by the passage of schistosome eggs through the intestinal epithelia, warrants their discussion in driving potential changes in microbiota, with associated shifts could have implications for the metabolome and its influence on immune priming (87). More generally, studies on the gut microbiome have yielded strong data on associated changes to metabolite profiles, including anti-inflammatory short-chain fatty acid (SCFA) profiles greatly implicated in gut-health-associated processes of immune tolerization (88). Direct changes to SCFA have been shown through *in utero* exposure to reduce offspring responsiveness to allergic airway inflammation, with mouse models showing this associated with additional changes to transcriptional profiles of Treg-relevant epigenetic pathways in fetal lungs (89). Additionally, metabolite profiles of urine from schistosome infected mice indicate changes driven by altered liver function (90) which warrant further investigation of metabolite-driven effects on offspring development, aside from potential changes associated with microbiota. Particularly, skewed expression of genes associated with placental production of steroid hormones during murine schistosome infection (71), and early evidence from a human study supporting that this may also be the case in humans (91), suggests metabolic changes associated with the glucocorticoid and hormonal axis that may be implicated in driving developmental changes in offspring leading to altered immunity.

## Conclusion

Continued exposure to the complex sets of foreign epitopes and antigenic stimuli present and secreted by helminths would ensure a constant challenge to the immune system during ontogenesis, combined with enhanced regulatory signaling via maternal cytokines during the key early-window during development. The resulting immune education, with the potential outcome of a highly trained network of regulatory immune processes and suppressive signals, may produce a pre-set, helminth specific immune memory that might either protect against immune sequelae of infection of offspring (e.g., schistosomiasis) or might lead to enhanced susceptibility (e.g., filariasis) to infection. Interestingly, the parasite would profit from either situation. These signals may also mitigate the response to immune challenge with, for example, otherwise strong allergens.

In terms of populations where helminth infection is endemic, these concerns must be considered in relation to not only interventions during and post- pregnancy, but also in the wider trend toward personalizing medicine in understanding how past and current microbial and parasitic exposures have led to individualized skewing or training of the immune system. This individual history of immunogenic exposures may be at the root of not only idiosyncratic responses to standard vaccine protocols, but also the appearance of inflammatory and immune disease, meaning that practical application of continued work in this area extends far beyond support of de-worming or controversial re-worming practices. As such, deeper understanding of how schistosomes and their compounds can manipulate the immune system could teach us more general lessons about fine control over immune responses. By examining these processes during the highly vulnerable *in utero* and early postnatal periods, we could gain mechanistic insight into the influence wielded by environmental exposures and interventions during these impressionable time points and subsequent outcomes for immune health.

## Author Contributions

ML wrote the manuscript, CP wrote sections of the manuscript. Both authors contributed to manuscript conception, revision, and approval of submitted version.

### Conflict of Interest Statement

The authors declare that the research was conducted in the absence of any commercial or financial relationships that could be construed as a potential conflict of interest.
